# Variation in Shrimp Allergens: Place of Origin Effects on Food Safety Assessment

**DOI:** 10.3390/ijms25084531

**Published:** 2024-04-20

**Authors:** Ryley D. Dorney, Elecia B. Johnston, Shaymaviswanathan Karnaneedi, Thimo Ruethers, Sandip D. Kamath, Karthik Gopi, Debashish Mazumder, Jesmond Sammut, Dean Jerry, Nicholas A. Williamson, Shuai Nie, Andreas L. Lopata

**Affiliations:** 1 Molecular Allergy Research Laboratory, College of Public Health, Medical and Veterinary Sciences, Australian Institute of Tropical Health and Medicine, James Cook University, Townsville, QLD 4811, Australiashaymaviswanathan.karnaneedi@my.jcu.edu.au (S.K.);; 2 Centre for Sustainable Tropical Fisheries and Aquaculture, College of Science and Engineering, James Cook University, Townsville, QLD 4811, Australia; 3 Centre for Food and Allergy Research, Murdoch Children’s Research Institute, Melbourne, VIC 3052, Australia; 4 Tropical Futures Institute, James Cook University Singapore, Singapore 387380, Singapore; 5 School of Public Health, University Centre for Rural Health, The University of Sydney, Sydney, NSW 2006, Australia; 6 Australian Nuclear Science and Technology Organisation, Lucas Heights, NSW 2234, Australia; 7 Centre for Ecosystem Science, The School of Biological, Earth and Environmental Sciences, University of New South Wales, Sydney, NSW 2052, Australia; 8 Bio21 Mass Spectrometry and Proteomics Facility, The Bio21 Molecular Science and Biotechnology Institute, The University of Melbourne, Melbourne, VIC 3052, Australia

**Keywords:** shellfish, crustacean allergens, food allergen proteomics, tropomyosin, mass spectrometric analysis of allergens, allergen ELISA

## Abstract

Due to the widespread use of shellfish ingredients in food products, accurate food labelling is urgently needed for consumers with shellfish allergies. Most crustacean allergen detection systems target the immunorecognition of the allergenic protein tropomyosin. However, this mode of detection may be affected by an origin-dependent protein composition. This study determined if the geographic location of capture, or aquaculture, influenced the allergenic protein profiles of Black Tiger Shrimp (*Penaeus monodon*), one of the most farmed and consumed shrimp species worldwide. Protein composition was analysed in shrimp from nine different locations in the Asia–Pacific by SDS-PAGE, immunoblotting, and mass spectrometry. Ten of the twelve known shrimp allergens were detected, but with considerable differences between locations. Sarcoplasmic calcium-binding protein, myosin light chain, and tropomyosin were the most abundant allergens in all locations. Hemocyanin-specific antibodies could identify up to six different isoforms, depending on the location of origin. Similarly, tropomyosin abundance varied by up to 13 times between locations. These findings suggest that allergen abundance may be related to shrimp origin and, thus, shrimp origin might directly impact the readout of commercial crustacean allergen detection kits, most of which target tropomyosin, and this should be considered in food safety assessments.

## 1. Introduction

Shellfish allergy has been an increasing health concern over the past decade. Over 2% of the general population is affected by food allergy to shellfish, with much higher prevalence in regions with high seafood consumption [1,2]. Allergy to shellfish is often lifelong, similar to peanut allergy, and it can cause severe allergic reactions including anaphylaxis, therefore presenting a serious health risk to the individuals affected [3]. More adults than children are affected by this food allergy, and shrimp seem to be the most implicated crustacean species [1,3,4,5]. In the Asia–Pacific region, the prevalence of self-reported shellfish allergy ranges from 1% in children to 7.7% in adults [2,4].

Shellfish is a general term used to label commonly consumed invertebrate species belonging to the phyla Crustacea as well as Mollusca. Several allergenic proteins have been identified and characterised on a molecular level; however, the muscle protein tropomyosin is considered to be a major allergen found in most crustaceans and molluscs. Despite tropomyosin being the most common allergen, patient sensitisation profiles to specific allergens have been found to differ geographically [4]. Tropomyosin does not seem to be the major allergen in Hong Kong, with only 41% sensitised individuals as compared to Thailand with 69% or to other regions with up to 90% [2,6].

Shellfish allergy management is currently limited to strict avoidance of all shellfish-containing food products; thus, affected consumers heavily rely on safe and correct food labelling. This can be a challenge due to the widespread use of crustacean ingredients in the formulation of food products. This is of particular importance for consumers of shellfish due to the fast growth of the aquaculture industry, global trade, and the consumption of different shellfish species. Over 180 countries implement mandatory allergen labelling, and shellfish, included in the ‘Big Eight’ food allergen groups, need to be declared on packaged food to alert consumers, as is mandated by the Codex Alimentarius United States and European Union [7]. The WHO estimates that by 2050 up to 50% of the world population will have some type of allergy. This growing problem is supported by increasing food recalls related to undeclared allergens due to mislabelling as well as cross-contamination during food processing [8].

Consequently, reliable detection and quantification of shellfish in processed food are essential for the health and safety of affected consumers [9]. Most commercially available test systems for quantifying crustacean allergens in food products target the allergen tropomyosin, as demonstrated in at least 10 of 15 commercial tests (Supplementary Table S1). This alpha-helical protein is central to muscle contraction and regulation, and for this reason, the primary structure of tropomyosin is highly conserved among crustaceans and, to a lesser extent, also molluscs. Tropomyosin (33–38 kDa) is highly stable to heat treatment, retaining allergenicity even after extensive cooking [10,11]. However, a total of twelve different crustacean allergens are currently registered with the World Health Organisation (WHO)/International Union of Immunological Societies (IUIS), and all are quantified in the current study.

Variations in allergen composition and abundance have been investigated in some selected plant foods, demonstrating large variance between varieties and the environmental locations they are grown in [12]. Large variations in the major allergen Pru p 1 were observed in different peach and nectarine varieties, with up to a 50-fold difference seen in allergen levels [13]. Furthermore, differences in allergen IgE binding have been demonstrated among different commercial soybean lines and between different growing locations in the US, supporting the view that genetic and environmental differences between varieties can account for allergen variation [14]. Moreover, evaluations of commercial peanut and lupin ELISA kits demonstrate that sensitivity and selectivity vary greatly, mostly due to the different protein targets used [15,16].

There is a lack of information on variability among allergens in animal-derived food sources. Recent proteomic studies on fish grown in aquaculture or caught in the wild have demonstrated variation in protein profiles. The major fish allergen parvalbumin seems to be more abundant in farmed wild gilthead sea bream (*Sparus aurata*) as compared to wild-caught specimens [17]. However, the inverse relationship was demonstrated in wild European sea bass (*Dicentrarchus labrax*), which had higher parvalbumin levels [18,19]. These quantitative differences in a major food allergen can directly impact the allergenicity of a food as well as food safety assessment. Currently, there is a lack of understanding of the range of variation in endogenous allergens in specific shellfish species, possibly impacting food safety assessment. While large variations in allergens have been demonstrated for different shellfish species, including shrimp [20] and crab [21], the intraspecies variability of allergen expression in shrimp between different origins has not been investigated.

The aim of the present study was to evaluate the impact of place of origin on the detection of the major allergen target tropomyosin, as well as eleven additional known crustacean allergens, in Black Tiger Shrimp (*Penaeus monodon*), one of the most farmed and consumed shrimp species worldwide. Characterising and comparing the allergen profiles of *P. monodon* from different geographical locations, as well as aquaculture and wild-caught specimens, may provide insight into the suitability of current crustacean food safety assessment tools.

## 2. Results

### 2.1. Protein Separation by SDS-Gel Electrophoresis

**Figure 1:**
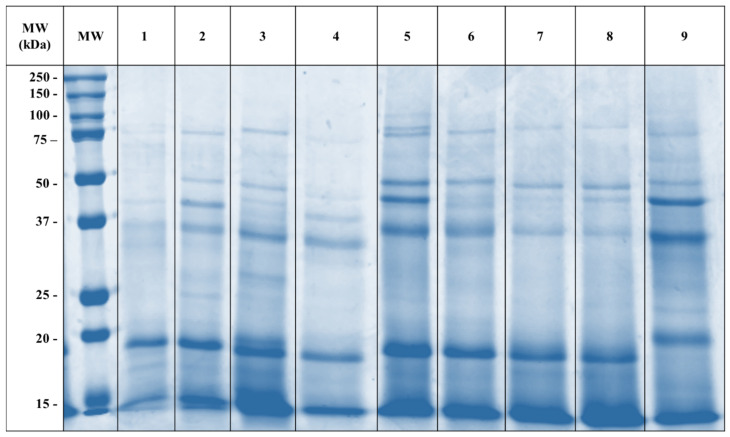
SDS-PAGE profiles of Black Tiger Shrimp samples (10 µg of protein from each sample) from nine different origins. 1 = Queensland-farmed; 2 = Queensland-wild caught; 3 = New South Wales-wild caught; 4 = Western Australia–wild caught; 5 = Indonesia-farmed; 6 = Indonesia-wild caught; 7 = China-farmed; 8 = China-wild caught; 9 = India-farmed specimen. Note: MW = molecular weight in kDa.

The protein composition of *P. monodon* specimen samples from nine different origins were compared by using SDS-PAGE (Figure 1). Distinct protein bands (<100 kDa) were visible in all extracts. While the total protein content was the same in all extracts, some showed more distinct protein bands, including the Indonesia-farmed and India-farmed specimens. The most abundant protein bands were between 35–50 kDa and 10–20 kDa.

### 2.2. Allergen Detection Using Allergen-Specific Antibodies

**Figure 2:**
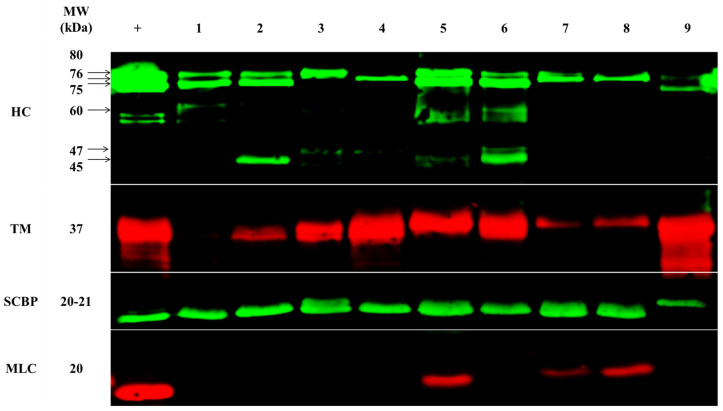
Allergen detection in shrimp samples of different origin by immunoblotting with allergen specific antibodies. + = Positive control (natural purified allergen); 1 = Queensland-farmed; 2 = Queensland-wild caught; 3 = New South Wales-wild caught; 4 = Western Australia—wild caught; 5 = Indonesia-farmed; 6 = Indonesia-wild caught; 7 = China-farmed; 8 = China-wild caught; 9 = India-farmed specimen. HC = hemocyanin, TM = tropomyosin, SCBP = sarcoplasmic calcium-binding protein, MLC = myosin light chain.

**Figure 3:**
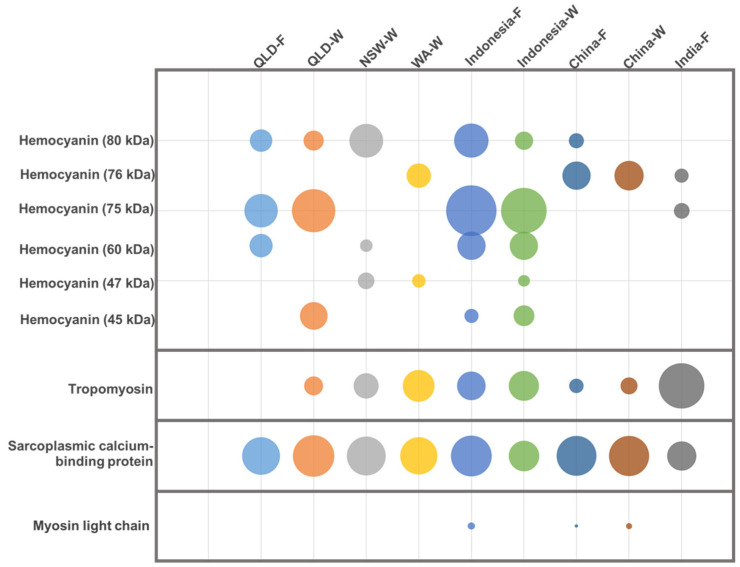
Densitometric analysis of differential binding intensity of allergen-specific antibodies from Figure 2 to hemocyanin isoforms, tropomyosin, sarcoplasmic calcium-binding protein, and myosin light chain in shrimp from different locations. QLD = Queensland; NSW = New South Wales; WA = Western Australia; F = farmed; W = wild caught. Size of bubble is indication of relative abundance.

Four WHO/IUIS-registered crustacean allergens, tropomyosin, HC, SCBP, and MLC were detected by using allergen-specific antibodies in most extracts (Figure 2 and Figure 3).

HC was detected in all extracts, with up to five bands in shrimp from Indonesia-wild specimen, and only one band in shrimp from Western Australia-wild, China-farmed/wild, and India-farmed specimen, followed by the 60 kDa band in Indonesia-wild and –farmed v, and the 45 kDa in Queensland- and Indonesia-wild specimen. The largest variation in antibody binding was demonstrated for HC, with up to six different bands recognised, ranging from 45 to 80 kDa. No single HC band was found across all samples, as each location had its own unique HC profile (e.g., China-wild specimen had a single band detected at 76 kDa, while Indonesia-wild specimen had all the observed bands except the 76 kDa band). The 75 kDa band was the most abundant where it was present, and the samples show variation in signal intensity of this band weight (i.e., Indonesia-farmed specimen had a 10-times stronger signal intensity compared to India-farmed specimen).

Tropomyosin was not detected by the allergen-specific antibody in Queensland-farmed shrimp, showed weak binding in Queensland-wild specimen, and China-farmed and -wild specimen, but strong binding to multiple bands in the remaining five samples. The highest signal intensity was seen in India-farmed shrimp, and this was 10-times greater than that to China-wild shrimp.

A single 20/21 kDa SCBP band was identified in all extracts; however, SCBP in India-farmed shrimp had a slightly higher molecular mass unique from all other locations. MLC was identified in only three of the nine extracts, revealing a weak band at 20 kDa in Indonesia-farmed shrimp and China-farmed and -wild shrimp.

### 2.3. Proteomic Analysis

**Figure 4:**
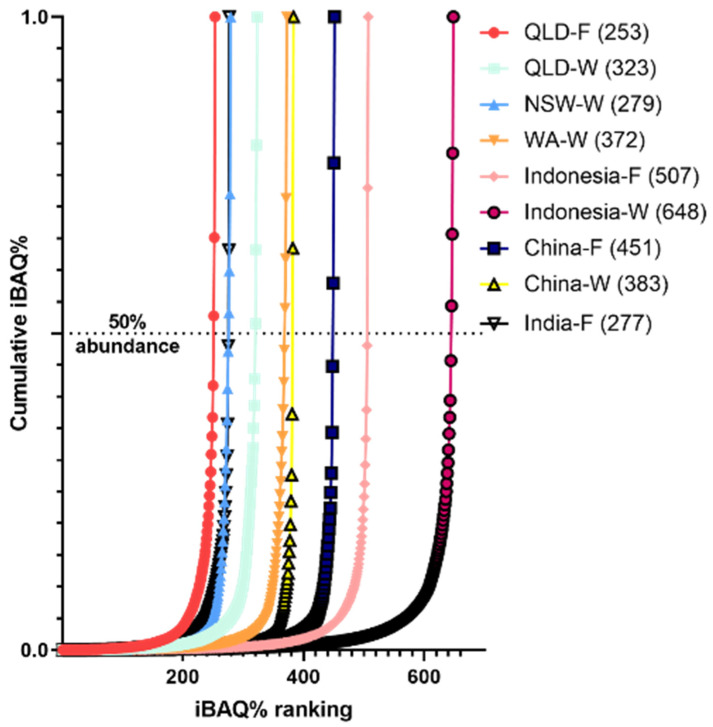
The cumulative iBAQ% from lowest to highest abundant proteins in relation to their contribution to the extract proteome from each provenance. iBAQ% is a measurement of relative abundance. Numbers in brackets indicate the number of proteins identified via mass spectrometry. QLD = Queensland; NSW = New South Wales; WA = Western Australia; F = farmed; W = wild caught.

The protein content in each of the nine sample groups were cross-validated using LC-MS proteomics/MS-based proteomic analysis. iBAQ intensities were used to determine their relative abundance, which varied greatly between the locations. The highest diversity of proteins was observed in Indonesia-wild shrimp (648 proteins identified) and the lowest was in the Queensland-farmed shrimp (253 proteins identified) (Figure 4).

Four major proteins, tropomyosin, MLC-1, MLC-2, and SCBP, contributed to more than 50% of all the identified proteins.

**Figure 5:**
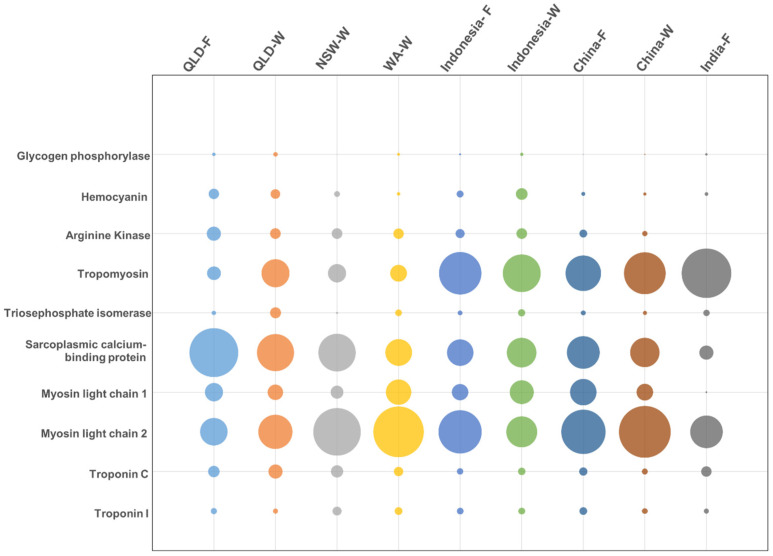
Relative abundance of known shellfish allergens in protein extracts for samples from each origin as estimated by using mass spectrometry analysis. Sizes of the bubbles indicate the relative abundance of the allergens calculated as an IBAQ%. QLD = Queensland; NSW = New South Wales; WA = Western Australia; F = farmed; W = caught.

In this study, we could identify ten of the twelve WHO-IUIS-registered allergens using mass spectrometry. To highlight the differences in the relative abundance of each identified allergen, their relative abundance (iBAQ%) was plotted, as reported in Figure 5. Variability in abundance was observed for several allergens and across different locations. India-farmed shrimp had the lowest relative abundance of allergens (59%), while China-wild shrimp had the highest, with 85% of proteins being identified as allergens.

The predominant allergenic protein for five of the nine locations was MLC-2, ranging from 11% (Queensland-farmed shrimp) to 40% (China-wild shrimp) (Figure 5).

SCBP was the predominant allergen in Queensland-farmed shrimp (36%), while tropomyosin was predominant in Indonesia-wild shrimp (22%) and India-farmed shrimp (37%). Up to 10% of proteins were identified as MLC-1. FABP was not detected in any of the samples. The remaining allergens, HC, AK, TPI, Tpn-C, and -I, were detected with a relative abundance of up to 3%. HC was most abundant in Indonesia-wild shrimp (2%) and least abundant in India-farmed shrimp (0.2%). Interestingly, while HC was one of the most antibody-binding allergens when analysing protein extracts with allergen-specific antibodies (see Figure 3), its actual protein abundance is very low. This comparative difference again emphasises that a protein of very low abundance can bind to allergen-specific antibodies very strongly, and this therefore needs to be incorporated in the risk evaluation of allergenicity and food safety based on allergen detection using antibody-based tests.

Between wild and farmed shrimp, there was little difference in the relative abundance of allergens, except for MLC-2, which was more abundant in wild shrimp, and for tropomyosin, which was more abundant in farmed shrimp. There were more noticeable differences when comparing the relative abundance of allergens in relation to the country of origin. MLC-2 in Queensland-farmed shrimp was present at 11%, but in other countries it ranged to up to 40%. Whilst SCBP was slightly more abundant in Australian shrimp, the relative abundance of tropomyosin was 3–12% lower compared to other countries, with the highest seen in India-farmed shrimp (37%).

### 2.4. Isoallergens

Within each allergenic protein group, allergens can be defined as isoallergens or variants (isoforms) depending on their amino acid sequence identity. Isoallergens are homologous allergens that share a similar molecular size, similar or identical biological function, and an amino acid sequence identity of at least 67% [22]. Each isoallergen may have multiple forms of highly identical sequences (>90% identity, typically differing in only few amino acids), which are referred to as variants (or isoforms) [22].

In this study, the allergen-specific antibody-binding profiles seen when using four specific antibodies demonstrated the presence of four important allergens in shrimp from all locations (Figure 2). However, clear differences in presence were demonstrated for MLC. Tropomyosin displayed apparent differences in abundance, while the multiple HC bands indicate there may be isoallergens of HC at various molecular weights [23]. Furthermore, SCBP in India-farmed shrimp had a slightly higher molecular mass unique from all other locations.

In support of the proteomic data presented, we recently analysed the complete transcriptome of *P. monodon*, identifying the presence of different isoallergens for tropomyosin and SCBP [20]. However, only one predominant isoform of tropomyosin was most abundant, explaining the more targeted antibody response to only a single band (Figure 2). Monoclonal antibodies recognise relatively small protein patches of about 10 amino acids on the target protein, and substitutions between similar proteins can therefore result in differential antibody-binding patterns. The two tropomyosin isoforms identified in the transcriptome have a pairwise identity of about 95%. However, when aligning the protein sequence of these two isoforms, it becomes clear that there are 14 substitutions within a narrow window of 38 residues (Supplementary Figure S1A). This specific protein region seems to contain the greatest variation in amino acid substitutions when aligning multiple tropomyosin sequences from nine different crustaceans (Supplementary Figure S1B).

A similar sequence alignment for the two SCBP isoallergens identifies a pairwise identity of 82% and four regions of non-conserved amino acids substitutions (Supplementary Figure S2). In contrast, the two allergenic proteins MLC-1 and MLC-2 were equally abundant in the transcriptome. Nevertheless, antibody binding to MLC in the current study was only demonstrated in samples from three of the nine locations. The slightly different molecular weight bands could be a reflection of the different MLC proteins, MLC-1 (18 kDa) and MLC-2 (20 kDa). Sequence alignment confirms that these two MLCs are very different proteins with only 16% pairwise amino acid identity [20].

## 3. Discussion

Undeclared allergens present a serious health risk to allergic consumers because the management of shellfish allergy relies on strict food avoidance and correct food labelling. However, the basis for labelling in over 180 countries is provided by the International Codex Alimentarius Commission, which explicitly specified in the “General Standard for the Labelling of Pre-packaged Foods” that crustacea and its subsequent products should always be declared [24,25]. The vast majority of commercial detection systems for the quantification of crustacean allergens in food products target the major allergen tropomyosin [26,27,28,29,30,31]. Therefore, variation in the abundance as well as presence of different isoforms of tropomyosin may impact the detection and quantification of crustacean allergens in food products, therefore impacting food safety assessment. This study sought to characterise the quantitative differences in the major allergen target tropomyosin as well as eleven additional crustacean allergens of *P. monodon* sourced from different origins. In addition, isoform and variant diversity and the relative abundance of allergens were also investigated.

The clinical relevance of different allergens seems to be very dependent on the demographics of sensitised individuals. Sensitisation to tropomyosin seems to be much lower in consumers from Asian countries; Wai et al. [32] suggest that this could be due to different eating habits and food processing. While Western populations mainly consume the muscle of shelled shrimp, East and Southeast Asian populations additionally consume other parts of shrimp, including the cephalothorax, which is rich in enzymatic proteins, as well as the allergen hemocyanin (HC) [32]. IgE reactivity to the major allergen tropomyosin can be as low as 40% in subjects from Hong Kong and as high as 90% in various studies from Europe. Also, IgE reactivity to other crustacean allergens can vary: AK, 48.3–51%; MLC-2, 37.9–57%; SCBP, 34.5–45%; Tpn-C, 17.2%; HC, 29–38%; FABP, 27% [2,6,33,34,35,36].

Transcriptomic-based approaches have shown that the expression of known crustacean allergens can vary between different shrimp species [20]. Whilst no studies have investigated intraspecies variation in allergen profiles in relation to the provenance of shellfish, similar studies on fish have identified different protein profiles between farmed and wild fish [17,18,19]. In the context of allergies, the major fish allergen parvalbumin was observed to be more abundant in farmed *Sparus aurata* compared to their wild counterparts [17]. In *Dicentrarchus labrax* farmed specimens, glyceraldehyde-3-phosphate dehydrogenase and aldolase were more abundant; in contrast, parvalbumin was less abundant compared to the wild specimens [18].

To support the differences in allergen presence and abundance, we performed quantitative mass spectrometry. In total, ten of the twelve registered allergens were detected and their abundance was compared between origins: tropomyosin, arginine kinase (AK), myosin light chain 1 (MLC-1), myosin light chain 2 (MLC-2), sarcoplasmic calcium-binding protein (SCBP), troponin C (Tpn-C), troponin I (Tpn-I), triosephosphate isomerase (TpI), and glycogen phosphorylase (GP) (see Supplementary Table S2).

In general, heating (cooking) shrimp will usually result in a considerable degradation of labile allergens (see Table S2), and very heat-stable allergens such as tropomyosin seem to dominate. Most of the detected allergenic proteins in our study are stable to denaturation during heating. In our study, protein extracts were dried at a relatively low temperature of 60 °C, and this seems to have resulted in the residual detection of labile allergens including arginine kinase, triosephosphate isomerase, and glycogen phosphorylase. The stability of different shrimp allergens or even their less characterised isoforms in low temperatures has not been well studied, but it is expected to impact allergen detection and quantification.

While MLC was detected in very small amounts in only three locations using specific antibodies through immunoblotting, this protein had an abundant representation across all locations, as determined by using mass spectrometric analysis. In addition, the MS analysis was able to further distinguish between MLC-1 and MLC-2. MLC-1 was most predominant in China-farmed shrimp, but was similarly abundant across all locations, and MLC-2 was most predominant in China-wild shrimp, with some variation across locations. Although immunoblotting was unable to detect tropomyosin in Queensland-F shrimp, mass spectrometry did detect tropomyosin, although with very low abundance. Similar to what was observed by immunoblotting, India-farmed shrimp had the highest relative abundance of tropomyosin, at nearly 13-times more compared to the lowest abundance in Queensland-farmed shrimp. Shrimp from Australia generally presented a lower tropomyosin abundance as compared to shrimp from other locations. There is little information to further elaborate on the mechanisms for the differences in the relative abundance of tropomyosin. Nevertheless, altered expressions of MLC-2 and tropomyosin have been associated with viral infection in two different shrimp species [37,38,39].

Data on the environmental conditions of the prawn specimens were not collected, and thus the current study is limited in providing direct explanations for the proteomic differences observed between provenances. Future studies would benefit from data on factors such as temperature, salinity, and oxygen levels, as these have been shown to influence the expression of some prawn proteins, including recognised allergens such as hemocyanin and myosin light chain [40,41]. As the current study’s aim was to compare allergenic protein profiles between origins as well as assess proteomic differences and their potential influence on food safety, genomic analyses were not performed on the prawns. Therefore, the genetic contribution to observed proteomic differences is unknown; however, prawns are likely to exhibit genetic diversity between locations. An analysis of SNPs and population structure by Vu et al. [42] suggests that geographically discrete *P. monodon* populations have likely undergone local adaptation to region-specific thermal regimes. Other studies on haplotype diversity also support *P. monodon* population differentiation between different locations in the Indo–Pacific region [42,43,44], and also between aquaculture and wild-caught prawns [45]. Whilst the current study does not have access to both wild-caught and aquaculture prawns from all locations, this is unlikely to greatly impact the findings of our study as aquaculture prawn populations are typically independent of their wild counterparts.

## 4. Materials and Methods

### 4.1. Sampling, Protein Extraction, and SDS-PAGE Analysis

Protein powder of individual specimens was prepared following the procedures described in detail in Gopi et al. [46]. In short, sixty-three specimens of *P. monodon*, sized 10 to 14 cm, were collected from farmed and wild-caught shrimp from three Australian states (Western Australia, New South Wales, and Queensland) and three Asian countries (China, India, and Indonesia) (Supplementary Figure S3). All specimens were immediately frozen upon collection and transported to the research facilities. The dissected muscle tissue was oven-dried at 60 °C for 48 h, and each individual specimen was subsequently homogenised into a fine powder. Powders from seven individual specimens for each location were pooled to generate one pooled sample for each of the nine locations. The generated protein powders were incubated in PBS (1 g powder per 4 mL PBS) overnight for 16 h at 4 °C, and this was followed by centrifugation at 20,000× *g* for 10 min. The resulting supernatant was filtered using a 0.45 μm filter and kept frozen at −80 °C until further use. The protein concentration of the extracts was determined using a Pierce™ BCA (bicinchoninic acid) Protein Assay Kit (ThermoFisher Scientific, Cambridge, MA, USA). Additionally, 10 µg of protein from each sample was separated by using sodium dodecyl-sulphate polyacrylamide gel electrophoresis (12% SDS-PAGE) along with Precision Plus Protein™ Dual Colour Standard (Bio-Rad, Hercules, CA, USA). Proteins were visualised by using Coomassie Brilliant Blue R-250 (Bio-Rad).

### 4.2. Immunoblotting with Allergen-Specific Antibodies

Following SDS-PAGE, proteins were transferred onto nitrocellulose membrane (Bio-Rad) and blocked with 1× Casein Blocking Buffer (Sigma-Aldrich, St. Louis, MO, USA) in PBS for 1 h. Membranes were then incubated on a rocking platform with primary antibodies in 0.5× Casein Blocking Buffer (Sigma-Aldrich) in PBS plus 0.1% Tween-20 (PBS-T) for 1 h. Four different allergen-specific antibodies were utilised: monoclonal anti-tropomyosin (ab50567) (Abcam, Cambridge, UK), in-house generated monoclonal anti-MLC and anti-SBCP raised in mice, and in-house polyclonal anti-HC raised in sheep. For each allergen analysed, 0.5 µg of the respective natural purified shrimp allergen was loaded as a positive control. Bound antibodies were detected with secondary antibodies by using IRDye^®^ 680RD and IRDye^®^ 800CW, (Li-Cor Biosciences, Lincoln, NE, USA), and the infrared signals were measured using the Odyssey^®^ CLx Imaging System (Li-Cor Biosciences). Data from the imaged immunoblots were imported into the Image Studio™ software (version 5.2.5) for analysis of the relative binding intensity. The fluorescence signal of each band was digitised, and the final intensity values were obtained by subtracting the background and blank control.

### 4.3. In-Solution Tryptic-Digestion

Here, 10 µg of protein in 5% SDS and triethylammonium bicarbonate (TEAB; 1 M) at pH 7.5 was processed by using an S-trap micro spin column (ProtiFi, Fairport, NY, USA). Proteins were reduced with 10 mM Tris (2-carboxyethyl) phosphine for 45 min at room temperature, followed by alkylation with 55 mM iodoacetamide in the dark for 45 min at room temperature. The resultant solution was centrifuged through the S-trap column at 4000× *g*, following the addition of 100 mM TEAB in 90% methanol at pH 7.5. Phosphoric acid was added to a final concentration of 1.2%. Proteins in the S-trap column were cleaned four times with 150 µL 100 mM TEAB in 90% methanol. The clean protein was then digested with 1 µg trypsin in 20 µL 50 mM TEAB at pH 7.5 overnight. Tryptic-digested peptides were sequentially eluted in 40 µL 50 mM TEAB at pH 7.5, 40 µL 0.2% formic acid, and 40 µL 50% acetonitrile at 4000× *g*.

### 4.4. Mass Spectrometry Analysis

Following the in-solution tryptic digestion, the samples were freeze-dried, resuspended in 2% ACN/0.05% trifluoracetic acid (TFA), and analysed via nanoESI-LC-MS/MS, as described previously [47]. In brief, the extracted peptides were analysed by using an LTQ Orbitrap Elite (Thermo Scientific) coupled to an Ultimate 3000 RSLC nanosystem (Dionex, Sunnyvale, CA, USA). The nanoLC system was equipped with an Acclaim Pepmap nano-trap column and an Acclaim Pepmap analytical column. The peptide mix was loaded onto the trap column before the enrichment column was switched in-line with the analytical column. The LTQ Orbitrap Elite mass spectrometer was operated in the data-dependent mode, and spectra were first acquired in positive mode at a resolution of 120,000 at *m*/*z* 200, and then by data-dependent HCD-MS/MS of precursor ions at a resolution of 15,000 at *m*/*z* 200.

### 4.5. Data Analysis

LC-MS/MS raw data were analysed by using MaxQuant (v. 1.6.17.0) against all 97,676 amino acid sequences for *Penaeus monodon* genus from the NCBI database [downloaded 1 January 2021; “Penaeus monodon” [porgn:__txid6687]]. The digestion enzyme was trypsin, and the allowed number of missed cleavages was 2. The variable modifications were methionine oxidation and protein N-terminal acetylation, and fixed modification was cysteine carbamidomethylation. The false discovery rate (FDR) at peptide spectrum match (PSM), and peptide and protein levels were set at 1%. All other settings were default values in MaxQuant. In the resultant protein group table, potential contaminant, reverse hit, and protein groups with less than 2 razor + unique peptides were removed in each sample. The relative protein abundance was calculated by applying intensity-based absolute quantification (iBAQ), which has been proven to have a good correlation with known relative protein amounts over at least four orders of magnitude [48]. This method estimates protein copy number as the sum of all tryptic peptides identified for each protein divided by the theoretically observable peptides obtained by in silico digestion, considering only peptides consisting of 6–30 amino acid residues. The relative protein abundance in each sample is expressed as a relative iBAQ value (iBAQ%).

## 5. Conclusions

The findings of this study suggest that the allergen profile of *P. monodon* displays considerable intraspecies differences, as shown by variability in the levels of allergenic proteins as well as isoforms between populations from different origins. Tropomyosin demonstrated up to 13-times difference in abundance between origins, while MLC was consistently abundant in all shrimp. In general, tropomyosin appeared to be more abundant in farmed shrimp, while MLC-2 was most abundant in wild shrimp. Because most commercial food allergen detection systems used on the food products for crustacean allergens target the protein tropomyosin, the results of this study show that the geographic origins of shrimp might directly impact the content level of this allergenic protein; thus, the readout of these commercial tests may be impacted and should be thoroughly considered in food safety assessment. Future studies should aim at comparing the allergenicity of *P. monodon* from various locations using patient IgE antibodies to determine if these differences in the abundance of allergens impact allergenicity and food safety.

## Data Availability

The original contributions presented in the study are included in the article/Supplementary Materials, and further inquiries can be directed to the corresponding author/s.

## Supplementary Materials

The supporting information can be downloaded at: https://www.mdpi.com/article/10.3390/ijms25084531/s1.

## References

[1] Khora S.S. (2016). Seafood-Associated Shellfish Allergy: A Comprehensive Review. Immunol. Investig..

[2] Ruethers T., Taki A.C., Johnston E.B., Nugraha R., Le T.T.K., Kalic T., McLean T.R., Kamath S.D., Lopata A.L. (2018). Seafood allergy: A comprehensive review of fish and shellfish allergens. Mol. Immunol..

[3] Goh S.H., Soh J.Y., Loh W., Lee K.P., Tan S.C., Heng W.J.K., Ibrahim I., Lee B.W., Chiang W.C. (2018). Cause and Clinical Presentation of Anaphylaxis in Singapore: From Infancy to Old Age. Int. Arch. Allergy Immunol..

[4] Moonesinghe H., Mackenzie H., Venter C., Kilburn S., Turner P., Weir K., Dean T. (2016). Prevalence of fish and shellfish allergy: A systematic review. Ann. Allergy Asthma Immunol..

[5] Warren C.M., Aktas O.N., Gupta R.S., Davis C.M. (2019). Prevalence and characteristics of adult shellfish allergy in the United States. J. Allergy Clin. Immunol..

[6] Wai C.Y.Y., Leung N.Y.H., Leung A.S.Y., Ngai S.M., Pacharn P., Yau Y.S., Rosa Duque J.S.D., Kwan M.Y.W., Jirapongsananuruk O., Chan W.H. (2022). Comprehending the allergen repertoire of shrimp for precision molecular diagnosis of shrimp allergy. Allergy.

[7] Food and Agricultural Organisation of the United Nations, World Health Organization (2018). General Standard for the Labelling of Prepackaged Foods. Codex Alimentarius: International Food Standards.

[8] Soon J.M., Abdul Wahab I.R. (2021). Global food recalls and alerts associated with labelling errors and its contributory factors. Trends Food Sci. Technol..

[9] Gelis S., Rueda M., Valero A., Fernandez E.A., Moran M., Fernandez-Caldas E. (2020). Shellfish Allergy: Unmet Needs in Diagnosis and Treatment. J. Investig. Allergol. Clin. Immunol..

[10] Faisal M., Vasiljevic T., Donkor O.N. (2019). Effects of selected processing treatments on antigenicity of banana prawn (*Fenneropenaeus merguiensis*) tropomyosin. Int. J. Food Sci. Technol..

[11] Wong L., Tham E.H., Lee B.W. (2019). An update on shellfish allergy. Curr. Opin. Allergy Clin. Immunol..

[12] Fernandez A., Mills E.N., Lovik M., Spoek A., Germini A., Mikalsen A., Wal J.M. (2013). Endogenous allergens and compositional analysis in the allergenicity assessment of genetically modified plants. Food Chem. Toxicol..

[13] Jin J., Gan K., Zhao L., Jia H., Zhu Y., Li X., Yang Z., Ye Z., Cao K., Wang Z. (2021). Peach allergen Pru p 1 content is generally low in fruit but with large variation in different varieties. Clin. Transl. Allergy.

[14] Lu M., Jin Y., Ballmer-Weber B., Goodman R.E. (2018). A comparative study of human IgE binding to proteins of a genetically modified (GM) soybean and six non-GM soybeans grown in multiple locations. Food Chem. Toxicol..

[15] Jayasena S., Smits M., Fiechter D., de Jong A., Nordlee J., Baumert J., Taylor S.L., Pieters R.H., Koppelman S.J. (2015). Comparison of six commercial ELISA kits for their specificity and sensitivity in detecting different major peanut allergens. J. Agric. Food Chem..

[16] Koeberl M., Sharp M.F., Tian R., Buddhadasa S., Clarke D., Roberts J. (2018). Lupine allergen detecting capability and cross-reactivity of related legumes by ELISA. Food Chem..

[17] Piovesana S., Capriotti A.L., Caruso G., Cavaliere C., La Barbera G., Zenezini Chiozzi R., Lagana A. (2016). Labeling and label free shotgun proteomics approaches to characterize muscle tissue from farmed and wild gilthead sea bream (*Sparus aurata*). J. Chromatogr. A.

[18] Monti G., De Napoli L., Mainolfi P., Barone R., Guida M., Marino G., Amoresano A. (2005). Monitoring food quality by microfluidic electrophoresis, gas chromatography, and mass spectrometry techniques: Effects of aquaculture on the sea bass (*Dicentrarchus labrax*). Anal. Chem..

[19] Chiozzi R.Z., Capriotti A.L., Cavaliere C., La Barbera G., Montone C.M., Piovesana S., Lagana A. (2018). Label-Free Shotgun Proteomics Approach to Characterize Muscle Tissue from Farmed and Wild European Sea Bass (*Dicentrarchus labrax*). Food Anal. Methods.

[20] Karnaneedi S., Huerlimann R., Johnston E.B., Nugraha R., Ruethers T., Taki A.C., Kamath S.D., Wade N.M., Jerry D.R., Lopata A.L. (2021). Novel Allergen Discovery through Comprehensive De Novo Transcriptomic Analyses of Five Shrimp Species. Int. J. Mol. Sci..

[21] Azemi N.F.H., Misnan R., Keong P.B., Yadzir Z.H.M. (2020). Reference gene and tropomyosin expression in mud crab Scylla olivacea, Scylla paramamosain and Scylla tranquebarica. Mol. Biol. Rep..

[22] Pomes A., Davies J.M., Gadermaier G., Hilger C., Holzhauser T., Lidholm J., Lopata A.L., Mueller G.A., Nandy A., Radauer C. (2018). WHO/IUIS Allergen Nomenclature: Providing a common language. Mol. Immunol..

[23] Stoeva S., Idakieva K., Georgieva D.N., Voelter W., Genov N. (2001). Penaeus monodon (tiger shrimp) hemocyanin: Subunit composition and thermostability. Z. Naturforschung C J. Biosci..

[24] Diaz-Amigo C., Popping B., Melton L., Shahidi F., Varelis P. (2019). Food Allergens: A Regulatory/Labelling Overview including the VITAL Approach. Encyclopedia of Food Chemistry.

[25] Mainente F., Fratea C., Simonato B., Zoccatelli G., Rizzi C. (2017). The Food Allergy Risk Management in the EU Labelling Legislation. J. Agric. Environ. Ethics.

[26] Li J., Wang H., Cheng J.H. (2021). DNA, protein and aptamer-based methods for seafood allergens detection: Principles, comparisons and updated applications. Crit. Rev. Food Sci. Nutr..

[27] Eischeid A.C., Stadig S.R., Rallabhandi P. (2021). Comparison of real-time PCR and ELISA for the detection of crustacean shellfish allergens. Food Addit. Contam. Part A Chem. Anal. Control Expo. Risk Assess..

[28] Jeong S.G., Kim S.H. (2020). Application of commercial kits using DNA-based and immunochemical methods for determination of shrimp allergens in kimchi and its ingredients. J. Food Sci..

[29] Zhou J., Wang Y., Qian Y., Zhang T., Zheng L., Fu L. (2020). Quantification of shellfish major allergen tropomyosin by SPR biosensor with gold patterned Biochips. Food Control.

[30] Wang Y., Li Z., Lin H., Siddanakoppalu P.N., Zhou J., Chen G., Yu Z. (2019). Quantum-dot-based lateral flow immunoassay for the rapid detection of crustacean major allergen tropomyosin. Food Control.

[31] Zhao J., Li Y., Li R., Timira V., Dasanayaka B.P., Zhang Z., Zhang J., Lin H., Li Z. (2022). Evaluation of poly- and monoclonal antibody-based sandwich enzyme-linked immunosorbent assay (ELISA) for their performance to detect crustacean residues in processed foods. Food Control.

[32] Wai C.Y.Y., Leung N.Y.H., Leung A.S.Y., Wong G.W.K., Leung T.F. (2021). Seafood Allergy in Asia: Geographical Specificity and Beyond. Front. Allergy.

[33] Pascal M., Grishina G., Yang A.C., Sanchez-Garcia S., Lin J., Towle D., Ibanez M.D., Sastre J., Sampson H.A., Ayuso R. (2015). Molecular Diagnosis of Shrimp Allergy: Efficiency of Several Allergens to Predict Clinical Reactivity. J. Allergy Clin. Immunol. Pract..

[34] Ayuso R., Sanchez-Garcia S., Lin J., Fu Z., Ibanez M.D., Carrillo T., Blanco C., Goldis M., Bardina L., Sastre J. (2010). Greater epitope recognition of shrimp allergens by children than by adults suggests that shrimp sensitization decreases with age. J. Allergy Clin. Immunol..

[35] Munera M., Martinez D., Wortmann J., Zakzuk J., Keller W., Caraballo L., Puerta L. (2022). Structural and allergenic properties of the fatty acid binding protein from shrimp *Litopenaeus vannamei*. Allergy.

[36] Liu M., Huan F., Li M., Han T., Xia F., Yang Y., Liu Q., Chen G., Cao M., Liu G. (2021). Mapping and IgE-binding capacity analysis of heat/digested stable epitopes of mud crab allergens. Food Chem..

[37] Li S., Li F., Sun Z., Zhang X., Xiang J. (2016). Differentially proteomic analysis of the Chinese shrimp at WSSV latent and acute infection stages by iTRAQ approach. Fish. Shellfish Immunol..

[38] Chongsatja P.O., Bourchookarn A., Lo C.F., Thongboonkerd V., Krittanai C. (2007). Proteomic analysis of differentially expressed proteins in Penaeus vannamei hemocytes upon Taura syndrome virus infection. Proteomics.

[39] Peruzza L., Shekhar M.S., Kumar K.V., Swathi A., Karthic K., Hauton C., Vijayan K.K. (2019). Temporal changes in transcriptome profile provide insights of White Spot Syndrome Virus infection in *Litopenaeus vannamei*. Sci. Rep..

[40] Shekhar M.S., Kiruthika J., Ponniah A.G. (2013). Identification and expression analysis of differentially expressed genes from shrimp (*Penaeus monodon*) in response to low salinity stress. Fish Shellfish Immunol..

[41] de la Vega E., Degnan B.M., Hall M.R., Wilson K.J. (2007). Differential expression of immune-related genes and transposable elements in black tiger shrimp (*Penaeus monodon*) exposed to a range of environmental stressors. Fish Shellfish Immunol..

[42] Vu N.T.T., Zenger K.R., Silva C.N.S., Guppy J.L., Jerry D.R. (2021). Population Structure, Genetic Connectivity, and Signatures of Local Adaptation of the Giant Black Tiger Shrimp (*Penaeus monodon*) throughout the Indo-Pacific Region. Genome Biol. Evol..

[43] Benzie J.A., Ballment E., Forbes A.T., Demetriades N.T., Sugama K., Haryanti, Moria S. (2002). Mitochondrial DNA variation in Indo-Pacific populations of the giant tiger prawn, Penaeus monodon. Mol. Ecol..

[44] You E.-M., Chiu T.-S., Liu K.-F., Tassanakajon A., Klinbunga S., Triwitayakorn K., de la Peña L.D., Li Y., Yu H.-T. (2008). Microsatellite and mitochondrial haplotype diversity reveals population differentiation in the tiger shrimp (*Penaeus monodon*) in the Indo-Pacific region. Anim. Genet..

[45] Xu Z.K., Primavera J.H., de la Pena L.D., Pettit P., Belak J., Alcivar-Warren A. (2001). Genetic diversity of wild and cultured Black Tiger Shrimp (*Penaeus monodon*) in the Philippines using microsatellites. Aquaculture.

[46] Gopi K., Mazumder D., Sammut J., Saintilan N., Crawford J., Gadd P. (2019). Combined use of stable isotope analysis and elemental profiling to determine provenance of black tiger prawns (*Penaeus monodon*). Food Control.

[47] Nugraha R., Kamath S.D., Johnston E., Zenger K.R., Rolland J.M., O’Hehir R.E., Lopata A.L. (2018). Rapid and comprehensive discovery of unreported shellfish allergens using large-scale transcriptomic and proteomic resources. J. Allergy Clin. Immunol..

[48] Schwanhausser B., Busse D., Li N., Dittmar G., Schuchhardt J., Wolf J., Chen W., Selbach M. (2011). Global quantification of mammalian gene expression control. Nature.

