# Supplementary material for: Variation in Shrimp Allergens: Place of Origin Effects on Food Safety Assessment
Source: Int J Mol Sci. 2024 Apr 20;25(8):4531. doi: 10.3390/ijms25084531 (PMC11050280; doi:10.3390/ijms25084531)
Supplement: Supplementary file 1 [file ijms-25-04531-s001.zip › ijms-2935889-supplementary.pdf]

Supplementary Table S1: Commercial Crustacea allergen detection kits. Described is each commercial kit's manufacturer, product name, the main analyte detected (Tropomyosin =TM; not specified = NS), technology used (ELISA = enzyme-linked immunosorbent assay; LFD = Lateral Flow Device) and Limit of Detection (LOD).

Manufacturer	Product	Main Analyte	Technology	LOD
Biosystems ES	Crustacean Rapid Test	TM	LFD	2.0 ppm
ELISASystems	Crustacean Residue Kit	TM	ELISA	NS
Eurofins Technologies	SENSISpec ELISA Crustaceans	TM	ELISA	NS
Eurofins Technologies	SENSIStrip Crustaceans	TM	LFD	NS
Hygiena	AlerTox ELISA Crustacean	TM	ELISA	1 ppm
Hygiena	AlerTox Sticks Crustacean	TM	LFD	3 µg
Morinaga	Crustacean ELISA Kit II	TM	ELISA	0.31 ppm
Morinaga	Rapid Test Easy for Crustacean	TM	LFD	0.5 ppm
R-Biopharm	RIDASCREEN®FAST Crustacean	TM	ELISA	2.0 ppm
Romer Labs	AgraQuant® Crustacea	TM	ELISA	20 ppm
3M	3M™ Crustacean Protein ELISA Kit	NS	ELISA	10.2 ppb
R-Biopharm	Bioavid Lateral Flow Crustacean	NS	LFD	10 ppm
Romer Labs	AgraStrip Crustacea	NS	LFD	2 ppm
Neogen	Reveal 3-D for Crustacea	NS	LFD	1 ppm
Neogen	Veratox® for Crustacea	NS	ELISA	<1 ppm

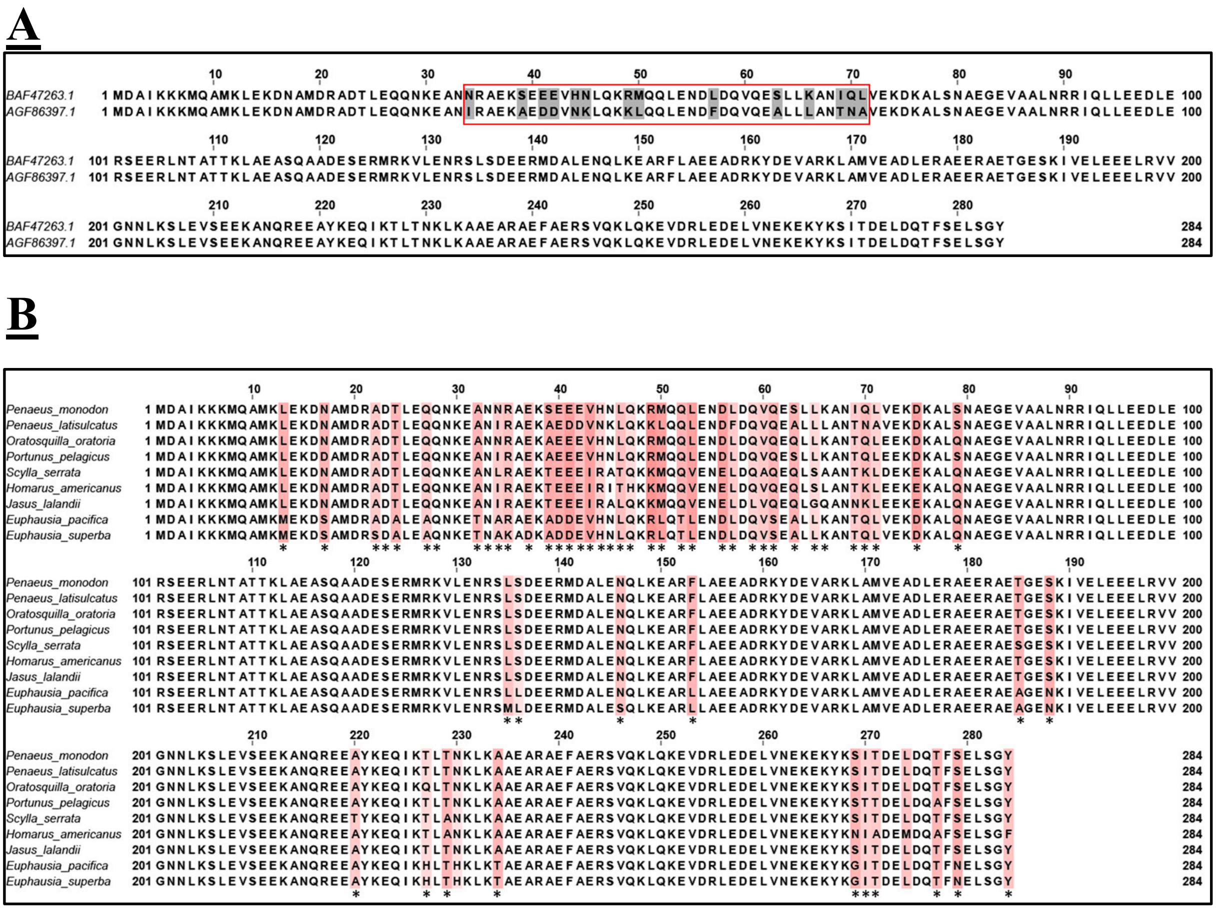

Supplementary Figure S1 (A) Pairwise sequence alignment of two isoforms of tropomyosin (TM) allergen identified within the transcriptome of *P. monodon*. Sequence names of isoforms are labeled with NCBI GenBank Accession number of the identified protein. Non-conserved amino acids are shaded in grey; regions of non-conserved amino acids are boxed in red. The two TM isoforms have a pairwise identity of 95%. Multiple sequence alignment was conducted in Jalview 2.11 using Clustal Omega. (**B)** Multiple sequence alignment of tropomyosin (TM) from 9 crustacean species. * denotes non-conserved amino acids and shades of red reflect level of conservation (darker red: less conserved). Multiple sequence alignment was conducted in Jalview 2.11 using Clustal Omega.

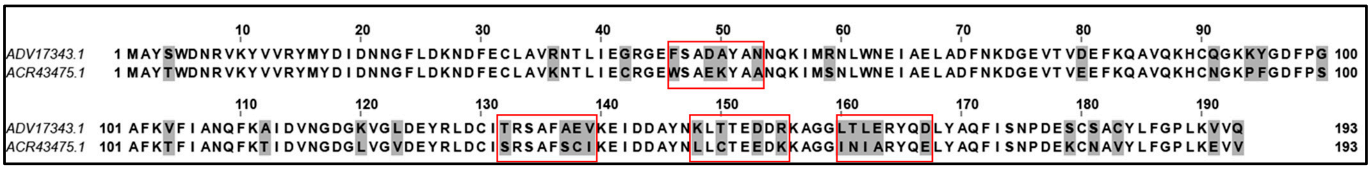

Supplementary Figure S2: Multiple sequence alignment of two isoforms of sarcoplasmic calcium-binding protein (SCBP) allergen identified within the transcriptome of P. monodon. Sequence names of isoforms are labeled with NCBI GenBank Accession number of the identified protein. Non-conserved amino acids are shaded in grey; regions of non-conserved amino acids (≥ 50%) are boxed in red. The two SCBP isoforms have a pairwise identity of 82%. Multiple sequence alignment was conducted in Jalview 2.11 using Clustal Omega.

Supplementary Table *S*2: WHO/IUIS registered allergenic proteins from Black Tiger Shrimp (*P. monodon*). Molecular Weight = MW (kDa). Adapted from Ruethers, Taki, Johnston, Nugraha, Le, Kalic, McLean, Kamath and Lopata [2]

WHO/IUIS Nomenclature	Function & Structure	MW (kDa)	Heat Stability	Ref.
	**Tropomyosin**			
Pen m 1	Alpha-helical coiled-coil dimeric protein. Controls the contraction of muscle fibres.	37	Highly stable	[2,47]
	**Arginine kinase**			
Pen m 2	Usually monomeric, it catalyses the reversible transfer of phosphoryl group from ATP to arginine	40	Labile	[2,47]
	**Myosin light chain**			
Pen m 3	Part of the larger myosin macromolecular complex that makes up the functional muscle protein structure. Functions in regulation of smooth muscle contraction, and is phosphorylated by MLC kinase.	20	Stable	[2,47]
	**Sarcoplasmic calcium-binding protein**			
Pen m 4	Contains helix-loop-helix motifs which bind calcium ions, and function in the regulation of calcium-based signalling. Crustacean SCBPs have two different subunits, α and β, which may form a homodimer or heterodimer that contains four EF-hand domains	20-25	Stable	[2,47]
	**Troponin**			
Pen m 6	A complex of three regulatory proteins involved in muscle contraction. Troponin C, the only subunit registered as an allergen in *P. monodon* regulates interaction of actin and myosin during muscle contraction on binding to calcium. Troponin I, registered as an allergen in Narrow-clawed crayfish, (*Pontastacus leptodactylus*) inhibits muscle contraction by binding to actin and preventing the actin-myosin interaction.	17 -21	Stable	[2]
	**Hemocyanin**			
Pen m 7	Forms hexamers of 450 kDa from a combination of 75 kDa structural subunits. Each subunit has two central copper-binding domains that perform the main oxygen transport function.	76	Stable	[2,47,48]
	**Triose phosphate isomerase**			
Pen m 8	Involved in glycolysis and gluconeogenesis. Converts D-glyceraldehyde 3-phosphate to dihydroxyacetone phosphate	28	Labile	[2]
	**Fatty acid binding protein**			
Pen m 13	Small cytosolic lipid-binding proteins responsible for transport of cytosolic long chain fatty acids and their metabolism.	15	Unknown	[33]

**Glycogen phosphorylase**				
Pen m 14	Cytosolic protein crucial in carbohydrate metabolism and involved in development and response to hormone stimulation and environmental stress. Predominantly expressed in brain and muscle	95	Unknown	[6]

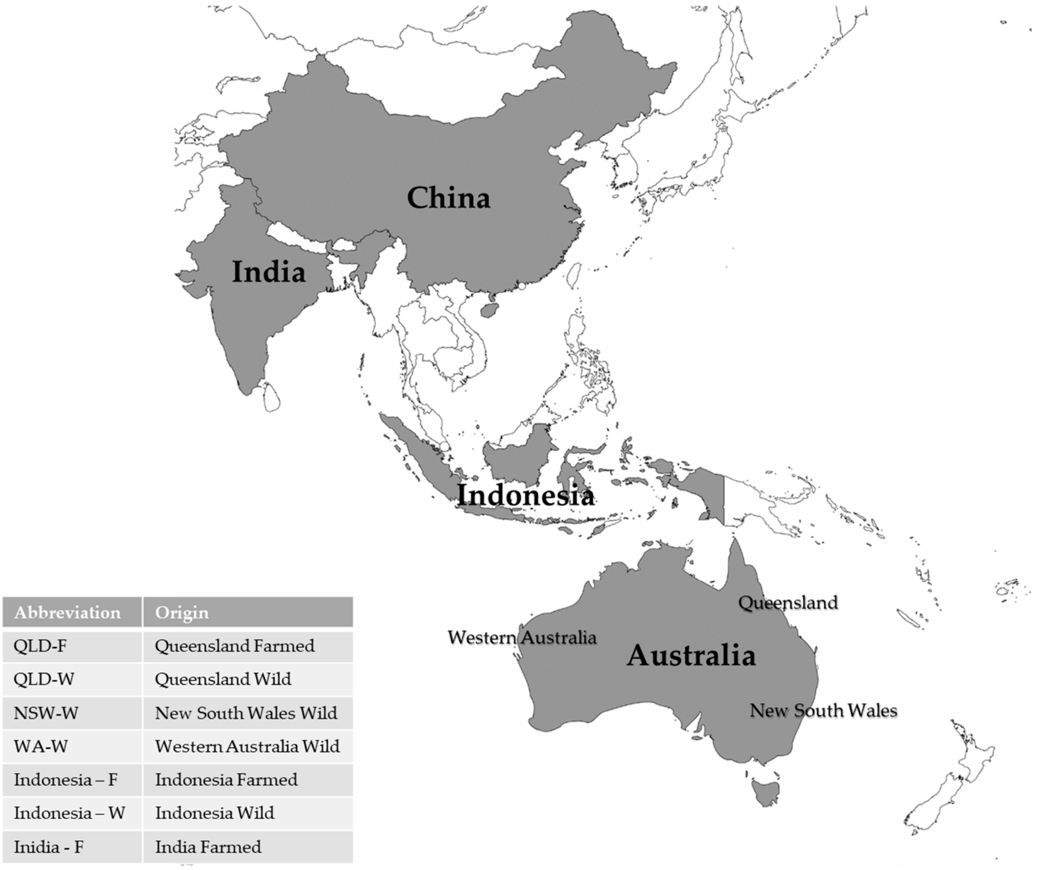

Supplementary Figure S3: Collection site of specimens in the Asia-Pacific. From each location seven farmed or wild *P. monodon* shrimps were collected, as described in Gopi, Mazumder, Sammut, Saintilan, Crawford and Gadd [44]
